# A wearable group-synchronized EEG system for multi-subject brain–computer interfaces

**DOI:** 10.3389/fnins.2023.1176344

**Published:** 2023-07-19

**Authors:** Yong Huang, Yuxiang Huan, Zhuo Zou, Weihua Pei, Xiaorong Gao, Yijun Wang, Lirong Zheng

**Affiliations:** ^1^School of Biomedical Engineering, Southern Medical University, Guangzhou, China; ^2^Brain-Inspired Computing Laboratory, Guangdong Institute of Intelligence Science and Technology, Hengqin, China; ^3^School of Information Science and Technology, Fudan University, Shanghai, China; ^4^Institute of Semiconductors, Chinese Academy of Sciences (CAS), Beijing, China; ^5^Department of Biomedical Engineering, Tsinghua University, Beijing, China

**Keywords:** group-synchronized, collaborative intelligence, multi-subject brain–computer interface, hyperscanning, wearable, real-time group-synchronized, real-time

## Abstract

**Objective:**

The multi-subject brain–computer interface (mBCI) is becoming a key tool for the analysis of group behaviors. It is necessary to adopt a neural recording system for collaborative brain signal acquisition, which is usually in the form of a fixed wire.

**Approach:**

In this study, we designed a wireless group-synchronized neural recording system that supports real-time mBCI and event-related potential (ERP) analysis. This system uses a wireless synchronizer to broadcast events to multiple wearable EEG amplifiers. The simultaneously received broadcast signals are marked in data packets to achieve real-time event correlation analysis of multiple targets in a group.

**Main results:**

To evaluate the performance of the proposed real-time group-synchronized neural recording system, we conducted collaborative signal sampling on 10 wireless mBCI devices. The average signal correlation reached 99.8%, the amplitude of average noise was 0.87 μV, and the average common mode rejection ratio (CMRR) reached 109.02 dB. The minimum synchronization error is 237 μs. We also tested the system in real-time processing of the steady-state visual-evoked potential (SSVEP) ranging from 8 to 15.8 Hz. Under 40 target stimulators, with 2 s data length, the average information transfer rate (ITR) reached 150 ± 20 bits/min, and the highest reached 260 bits/min, which was comparable to the marketing leading EEG system (the average: 150 ± 15 bits/min; the highest: 280 bits/min). The accuracy of target recognition in 2 s was 98%, similar to that of the Synamps2 (99%), but a higher signal-to-noise ratio (SNR) of 5.08 dB was achieved. We designed a group EEG cognitive experiment; to verify, this system can be used in noisy settings.

**Significance:**

The evaluation results revealed that the proposed real-time group-synchronized neural recording system is a high-performance tool for real-time mBCI research. It is an enabler for a wide range of future applications in collaborative intelligence, cognitive neurology, and rehabilitation.

## 1. Introduction

In recent years, mBCI and collaborative intelligence have gained great attention in the field of brain science (Czeszumski et al., 2020; Gao et al., 2021). Moreover, implementing a collaborative acquisition system, as a key tool for collaborative intelligence, is a fundamental problem in generalized BCI (Babiloni and Astolfi, 2014; Perdikis et al., 2020; Zhang et al., 2020; Bhattacharyya et al., 2021). Collaborative adaptive learning of AI requires human–human and human–machine collaboration in a synchronous manner with real-time access to relevant event information (Shenoy et al., 2014). Therefore, humans and machines can cooperate in an adaptive (Müller et al., 2017) and dynamic and effective way (van den Bosch et al., 2019). There are many examples of how the multi-brain works, for example, studies on predicting marital relationships through neural synchronization in multiple brains (Li et al., 2022) and effective interaction between teachers and students (Maksimenko et al., 2018). These investigations assist researchers in comprehending social cognition (Konvalinka and Roepstorff, 2012) and exploring the concept of the “Social Brain” (Minagawa et al., 2018). In addition, some collaborative approaches have demonstrated an mBCI that fused event-related potential (ERP) data for collective decision-making (Wang and Jung, 2011).

To support the development of collaborative mBCI applications, there is an increasing demand for an integrated system that encompasses multiple-target signal acquisition, synchronized triggering, and user-friendly configuration. When designing this system, we should consider the following: First, in non-laboratory mBCI experiments, external noise can interfere with the acquisition of system data, leading to a reduction in the signal-to-noise ratio (SNR) of EEG data and a decrease in data reliability. The desired mBCI system should provide high-quality EEG signals to guarantee its reliability and robustness when facing diverse ambient noise. Second, the traditional fixed-linked EEG devices are connected by cables to form a multi-subject acquisition system (Barraza et al., 2019). This fixed-linked system imposes limitations on subjects' range of motion and activities, consequently affecting their overall user experience and restricting the wider application of mBCI. Therefore, wireless-connected mBCI systems will be more favorable and offer significant advantages in future applications. Third, event-triggered synchronous signal acquisition is crucial for mBCI systems, as EEG signals gathered from different BCI devices require strict synchronization for further correlation analysis. For example, hybrid EEG and EMG synchronous acquisition (Artoni et al., 2018) and event-related potential (ERP) mechanisms with up to 100 classifications (Xu et al., 2020) have been implemented. This research highly requires low latency, synchronized phase (Xu et al., 2018), and time alignment of the EEG data. Time-space synchronization necessitates a few milliseconds or even <1 ms (Luck, 2014). Moreover, asynchronism may lead to incorrect estimates, such as time-domain correlation (Bowyer, 2016) and imaginary part correlation (Ayrolles et al., 2020). Traditional synchronization methods employ a wired trigger box as an event source for distributing synchronization signals, and some methods employ multi-device timestamps. Typically, there are two common synchronization methods: wired hardware synchronization and software synchronization. Hardware synchronization involves inputting a synchronization signal into a digital port (David Hairston et al., 2014). Pulse signals are typically generated by serial/parallel ports or sensors. For instance, audio signals are employed for synchronization (Pérez et al., 2021), and clock signals are sent to two wired acquisition devices (Chuang et al., 2021). On the other hand, software synchronization relies on programs that do offline calibration by aligning the data from multiple sources with timestamps in some protocols, such as the LSL (lab streaming layer) framework (Reis et al., 2014) or video frame synchronization (Raghavan et al., 2018).

In this study, we developed a wearable real-time group-synchronized EEG acquisition system to overcome the aforementioned challenges of an mBCI system. The proposed system integrates a light-based event trigger, wireless EEG acquisition devices, an analysis system, and an ERP stimuli system. Up to 10 wireless EEG acquisition devices can be group-synchronized by the event trigger, freeing the limitations of subjects' range of motion and activities. In addition, we optimized the wireless communication channels and the data packet protocol. The EEG acquisition subsystem achieved an average noise amplitude of 0.87 μV, a CMRR of 109.02 dB, a higher SNR, and a comparable ITR with the Synamps2 EEG system from the market-leading company Neuroscan. Finally, the effectiveness of the proposed mBCI system was verified in a multi-subject cognitive experiment, demonstrating its potential in research on social interaction and decision-making in cognitive neurology.

## 2. Materials

### 2.1. System architecture

**Figure 1:**
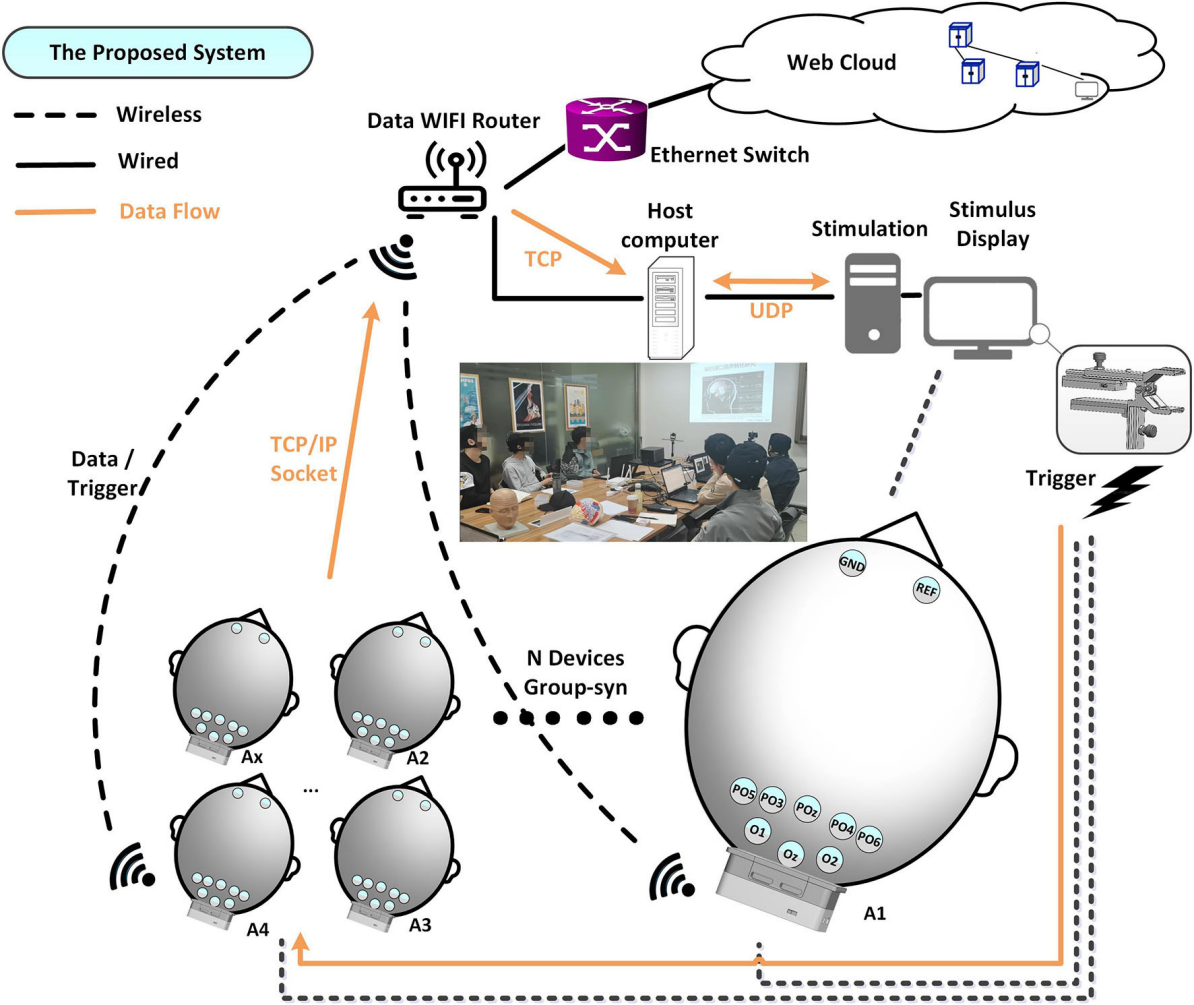
The proposed mBCI system implemented on 10 amplifiers.

The architecture of the proposed mBCI system is illustrated in Figure 1. The mBCI hardware system features a wireless trigger for data synchronization, a Wi-Fi router for wireless network connectivity, 10 wearable compact wireless amplifiers (A1–A10) at a 1 kHz sampling rate and a host computer for recording and analysis purposes. The system utilizes the light as the trigger source to send events simultaneously to all the wireless EEG amplifiers. EEG Ag/AgCl electrodes (PO6, PO4, POz, PO3, PO5, O1, Oz, and O2), along with one reference and one ground, are linked to the forehead area, as shown in Figure 1. Furthermore, we designed a group EEG cognitive experiment to verify that this system is effective and can be used in noisy settings.

**Figure 2:**
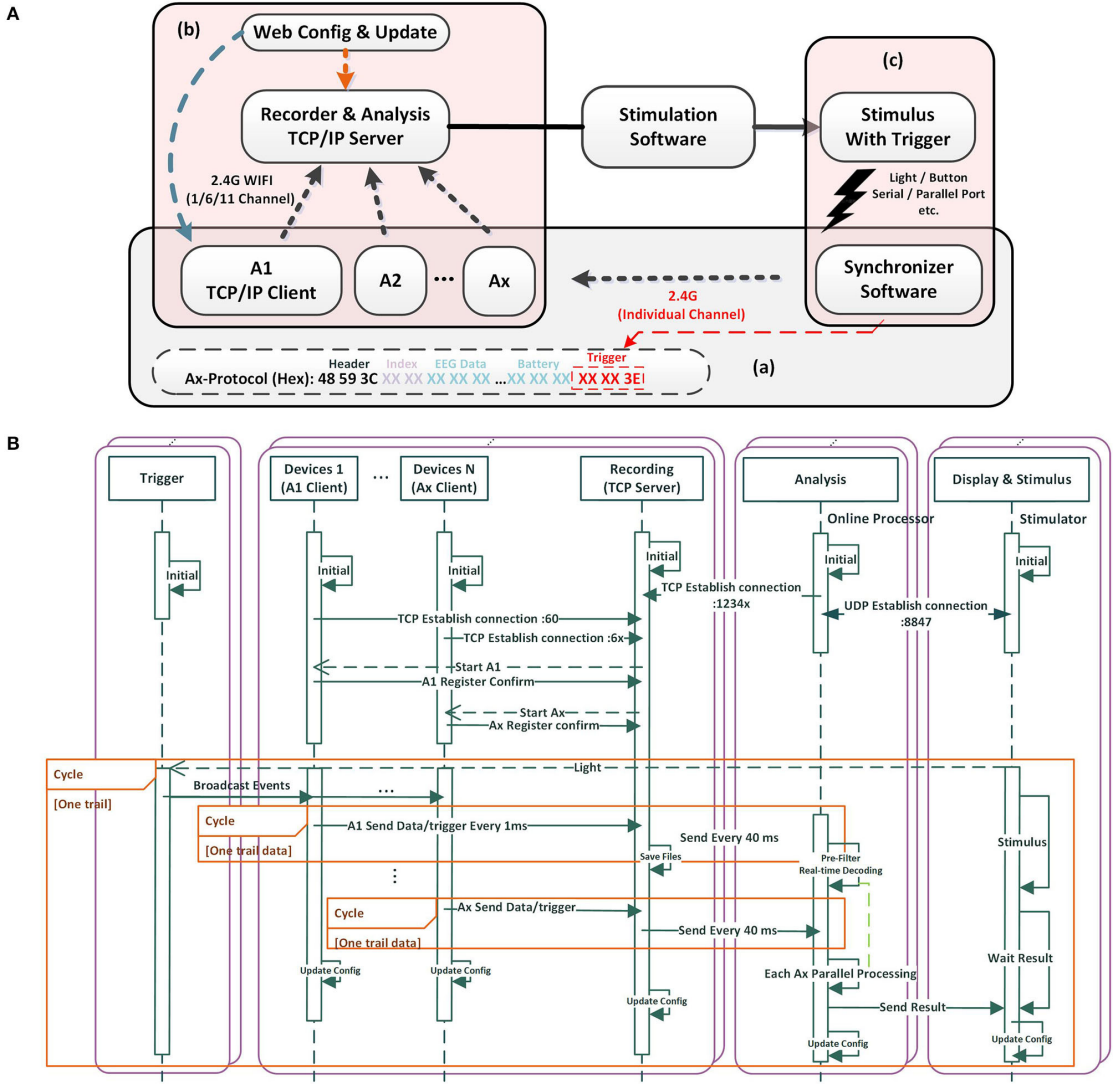
The mBCI system software architecture and processing flow. **(A)** Software featured with individual connection, run-time configuration and custom protocol. **(B)** The software processing flow.

The stimulus display shows real-time calculation results, stimulus signals, and optical synchronization signals, which are generated by a steady stimulus-producing host computer. A light sensor trigger is employed instead of alternative sensors, such as audio, to reduce latency and efficiency. The synchronizer processes the trigger signal and uses a dedicated 2.4G channel to transmit the output to the amplifiers, which differs from the channel between the recorder analysis server and the client A_x_. The interference may occur due to adjacent channel interference from the neighbor's wireless local area network channels in the 802.11 band devices. To ensure minimal interference between different channel signals, we processed the individual channels. The protocol of each device enables simultaneous marking of the received trigger signals (to indicate different events or sequences), as shown in Figure 2A(a) red box.

Raw EEG data packed together with the synchronized trigger event are wirelessly sent to the recording host computer (acquisition server) via TCP/IP socket. The host computer receives data packets from the devices and parses them according to the predefined data protocol. The recorder and analysis perform processing functions, such as filtering, data saving, and real-time decoding.

### 2.2. Software architecture

The software architecture is divided into four parts: trigger, recorder, analysis, and display of stimuli. The trigger is independent of the acquisition system and does not require an added hardware trigger box for connectivity. To ensure complete data are transmitted wirelessly, a TCP connection links the acquisition and recording software. Different algorithmic microservices acquire potential data from different devices over different ports when the data need to be simultaneously analyzed. TCP is used where reliable transmission is necessary at the transport layer, whereas UDP is used for communication where high-speed transmission and real-time performance are required. Because the quantity of data is minimal and resides under wired local area networks or local hosts, we used a UDP connection between display and analysis software. The UDP packet header is 8 bytes, with relatively low overhead compared with the 20-byte packet header in TCP. In addition, using UDP packets allow for lower connection latency and network traffic by reducing the three-way handshake. In this way, the results can be timely feedback to the display. Using a 40 ms packet transfer (35 bytes/ms, 1,400 bytes per packet), we maintained each maximum packet size within the maximum transmission unit (MTU) range of 1,500 bytes, thereby facilitating optimal TCP and UDP transfers. In brief, TCP is more intricate, with a higher volume of header data, which guarantees wireless communication reliability. Conversely, UDP saves network traffic by eliminating the requirement for packet loss retransmission, resulting in improved real-time performance.

We designed the application layer protocol, as shown in Figure 2A(a). A frame header (48 59 3C, 3 bytes) is used to locate different nodes. An index (from 00 00 to FF, 2 bytes) is used by the node to verify the data integrity. Each channel of EEG data consists of 3 bytes, while an additional 3 bytes are allocated for trigger data, which serves as event number for different events. Similarly, 3 bytes are assigned for battery information. Each packet contains 35 bytes, with a transmission rate of 1,400 bytes every 40 ms. The entire system can be run-time configured by updating the JSON file that defines the IP, port, channel, sample rate, and other variables. This feature allows for easy configuration updates while the system is running. In addition, the server and client A_x_ can be configured and updated by internet users, allowing upload data or reporting of results to some network infrastructure, as shown in Figure 2A(b). Based on the task design and stimulation software, the stimulus with trigger can be light, audio, button, serial/parallel port, and so on, as shown in Figure 2A(c).

According to the software processing flow (refer to Figure 2B), we prioritized server and client operations to secure efficient acquisition and recording. We designed the recording part as a TCP server. Once turned on, each device (client A_x_) automatically connects with the server, registers, and awaits the start acquisition command. Upon receiving the command, the client A_x_ sends the EEG data to the server. To further enhance data reliability, the recording unit opens an additional server port, awaiting a TCP connection from the analysis component. Once initiated, the analysis unit connects with the recording unit and establishes a UDP connection with the stimulus display. Additionally, the analysis part can operate independently without any online requirements.

To efficiently synchronize data, the display stimulus unit emits light. When detecting a sudden flash, the light trigger sends the synchronization signals to the devices through an independent channel. The synchronization signals can be marked in the data protocol packets of each device. Client A_x_ sends data/trigger to the recording TCP server every 1 ms. The recording TCP server sends to the analysis unit after packetizing every 40 ms. This process is repeated in subsequent rounds based on the experimental paradigm design.

## 3. Methods and experiments

We used a three-step method to evaluate the system's performance. The first step involved evaluation of hardware performance, the second involved evaluating ERP signal qualities, and the last involved conducting group cognition experiments.

### 3.1. Evaluation of hardware performance

We evaluated the hardware's performance based on three key parameters: data correlation coefficient, CMRR, and noise. In noisy settings, external industrial frequency and wireless interferences often cause common mode noise, which can disturb the acquired device data. In cases where physiological signals are weak, CMRR serves as a critical metric for demonstrating the ability to suppress common mode signals.

#### 3.1.1. Synchronization test

We used Pearson's correlation coefficient, as described by Rodgers and Nicewander (1988), to estimate the correlation between two waves in our study. The coefficient is defined as the quotient of covariance and standard deviation between two variables:


(1) $$\begin{array}{l} \rho_{X,Y}=\frac{\mathrm{cov}\left(X,Y\right)}{\sigma_{X}\sigma_{Y}}=\frac{E\left[\left(X-\mu_{X}\right)\left(Y-\mu_{Y}\right)\right]}{\sigma_{X}\sigma_{Y}} \end{array}$$

The abovementioned equation defines the overall correlation coefficient. For multi-channel data, we established the mutual covariance matrix using this equation.

**Figure 3:**
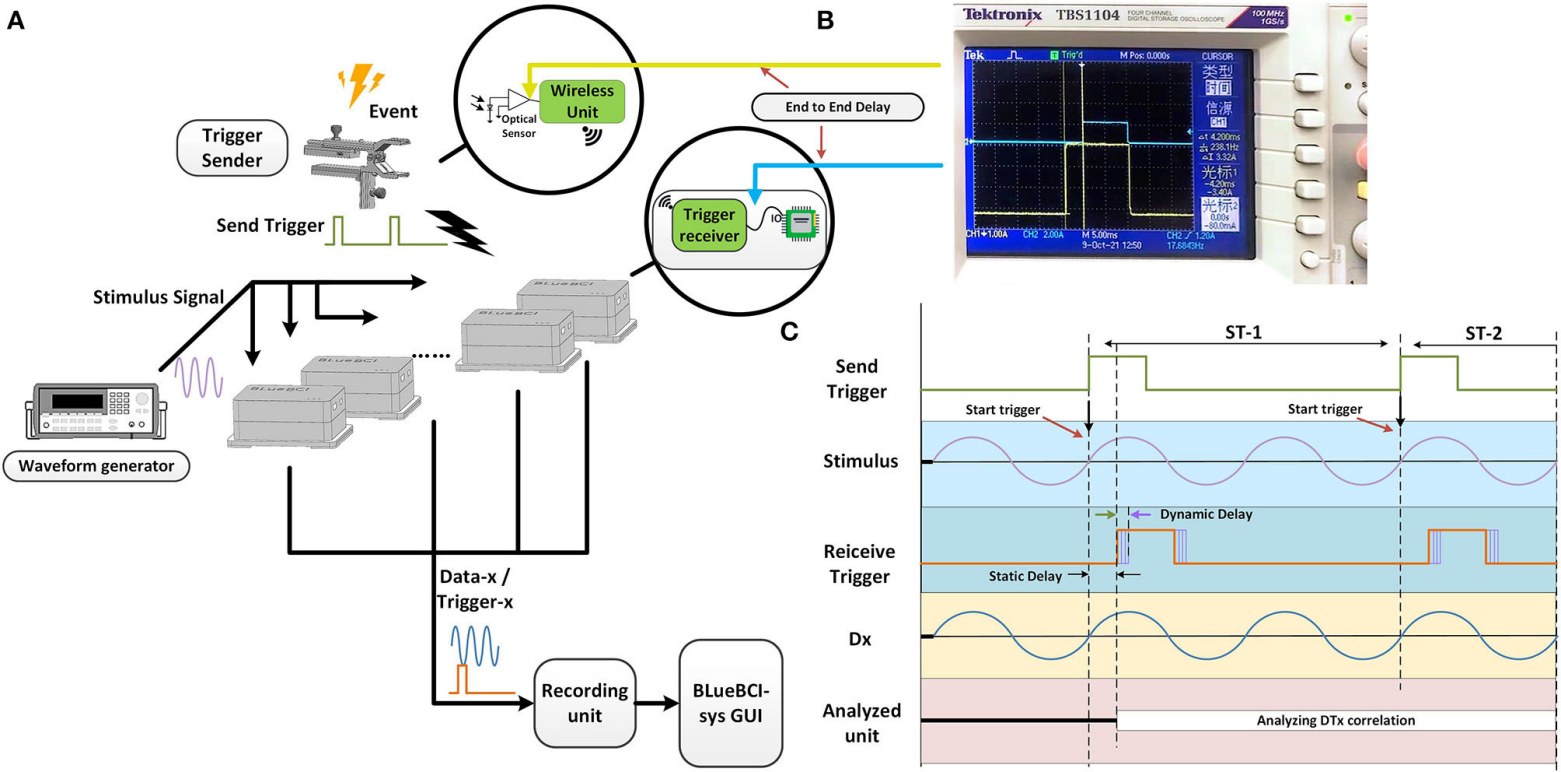
The mBCI system synchronization test. **(A)** The architecture of synchronization test. **(B)** The test of end-to-end delay. **(C)** The process of synchronization test and two types of the delays defined.

A light-trigger method was applied to test synchronization strategies by employing a signal generator and wireless trigger unit. Specifically, the signal generator sent out a stimulus signal, 10-Hz sine with 10 mVpp, as shown in Figure 3A. The signals were sent to each amplifier using a high-performance cable and lasted for 120 min. Throughout the continuous operation, we recorded and monitored the stability of signals using GUI software and inspected the output waveform of 10 devices every half an hour from an oscilloscope. Furthermore, the spacing between trigger occurrences was measured.

The trigger-sender contains an optical sensor. The wireless trigger-sender simultaneously sends the signal to the trigger-receiver of each EEG device, as shown in Figure 3A. In the experiment, the signal source and the 10 devices were positioned on opposite sides.

Time differences were measured to estimate interference caused by the spatial transmission between the trigger sender and receiver unit, as well as to examine time delays between each transceiver protocol. The end-to-end delay is the delay between the two probes, as shown in Figure 3B, including the static delay and the dynamic delay. We defined two types of delays, as shown in Figure 3C. The minimum synchronization error is the minimum value of the dynamic delay among the values obtained from repeated experiments.

#### 3.1.2. CMRR test

The CMRR indicates the rejection ability of the common mode signal in the differential amplifier. The calculation method is as follows:


(2) $$\begin{array}{l} CMRR=10\times \log_{10}{\left(\frac{V_{d}}{V_{cm}}\right)}^{2}=20\times \log_{10}\left(\frac{V_{d}}{V_{cm}}\right) \end{array}$$

where *V*_*d*_ represents the voltage amplification factor of the differential mode signal and *V*_*cm*_ represents the voltage amplification factor of the common mode signal. To measure the root mean square of the input noise, we short-circuited each input channel with the reference electrode. We employed this approach in the following experiments of hardware evaluation. To carry out the CMRR test, we connected all input channels and REF port to the positive output of the waveform generators, while connecting GND to the negative output. The waveform generators transmitted a 10-Hz sine wave with 500 mVpp through high-performance cable. We calculated the noise by shorting each input channel and the REF port with no signal.

**Figure 4:**
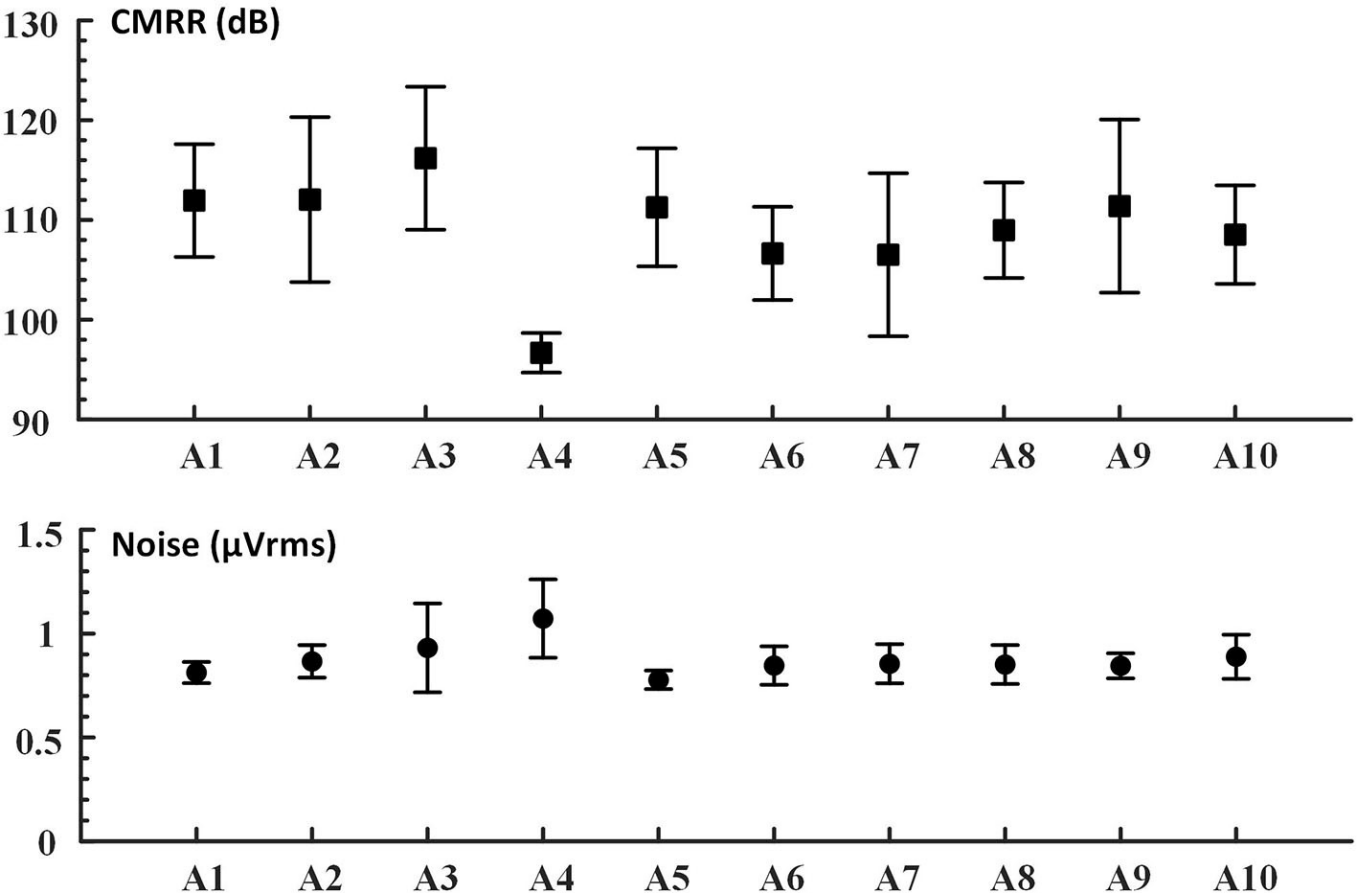
The device CMRR (dB) @10 Hz; noise (μVrms) @2–45 Hz. In this study, based on the mBCI 10 devices (A1–A10), the bandpass filters (2–45 Hz) for noise calculation from original EEG signals were in Chebyshev type I filter order. Evaluations were performed at 1 KHz sampling rates.

We proposed the novel mBCI system, each wearable compact amplifier weighs 56 ± 4 g, and the size is 59.3 × 47.4 × 22.7 mm. The average noise amplitude is 0.87 μVrms @2–45 Hz, and the average CMRR of all the tested devices except abnormal A4 is 109.03 dB @10 Hz, as shown in Figure 4. This wearable compact system allows a 10 m distance between users by hardware-based synchronization among 10 users. The receiver in each amplifier receives triggers/markers from the wireless trigger sender.

Typically, the noise evaluated in such cases is the input reference noise (Bolatkale et al., 2014). To overcome the motion and the high-frequency thermal noise of the components, we applied a filter that allowed signals from 2 to 45 Hz. One of the devices (A4) had a negligible deficit.

### 3.2. Functionality evaluation

For the functionality of the system, we selected the SSVEP method as the key indicator. SSVEP is capable of detecting the periodic synchronization of the brain with an external flickering visual stimulus delivered at a fixed frequency, making it an ideal tool for assessing synchronization accuracy and reliability. We assessed both the phase and frequency accuracy of EEG data recorded at the millisecond level. To compare the proposed system with the Synamps2 EEG system from the market-leading company Neuroscan, Inc., we designed both forty-targets and single-target SSVEP spelling experiments.

#### 3.2.1. SSVEP method

The method was previously described for visual spellers using the sampled sinusoidal stimulus method in a monitor stimulus (Manyakov et al., 2013; Chen et al., 2014). The modulation of the screen brightness represents a stimulus sequence corresponding to the frequency *f*.


(3) $$\begin{array}{l} s\left(f,i\right)=\;\frac{1}{2}\times \left\{1+sin\left[2\pi f\left(i/Refresh\;Rate\right)\right]\right\} \end{array}$$

where *sin*() generates a sine wave and *i* represents the frame index in the stimulus sequence. represents the Refresh Rate of the screen (the monitor or display).

We followed the method (Chen et al., 2015a,b; Wong et al., 2020) and applied filter bank canonical correlation analysis (FBCCA). This method is widely used to detect the frequency of SSVEP. The SNR and classification analysis can be used to evaluate SSVEP data (Chen et al., 2015a,b; Liu et al., 2020; Ladouce et al., 2022). In this paper, the SNR can be defined as follows:


(4) $$\begin{array}{l} SNR=20\;\log_{10}\frac{y\left(f\right)}{\frac{1}{2n}\cdot \overset{n=4}{\underset{k\text{=}1}{\sum }}y\left(f-\text{Δ}f\cdot k\right)+y\left(f+\text{Δ}f\cdot k\right)} \end{array}$$

where *y*(*f*) represents the spectrum calculated by fast Fourier transform, and Δ*f* represents the frequency resolution.

The recognition accuracy and ITR were defined (Chen et al., 2015a,b; Wang et al., 2017). The ITR represents the output information per second or minute. The calculation formula is as follows:


(5) $$\begin{array}{l} ITR=60\cdot \left({log}_{2}\text{N}+Plog_{2}P+\left(1-P\right)log_{2}\frac{1-P}{N-1}\right)/T \end{array}$$

The N is defined as the number of commands that can be output by the system. The accuracy of target recognition (P) affects the feasibility and reliability of the BCI communication system. Single-target selection time (T) is often defined as the time required for the BCI system to output a single command. This study refers to visual gaze duration in SSVEP experiments.

#### 3.2.2. SSVEP experiments

**Figure 5:**
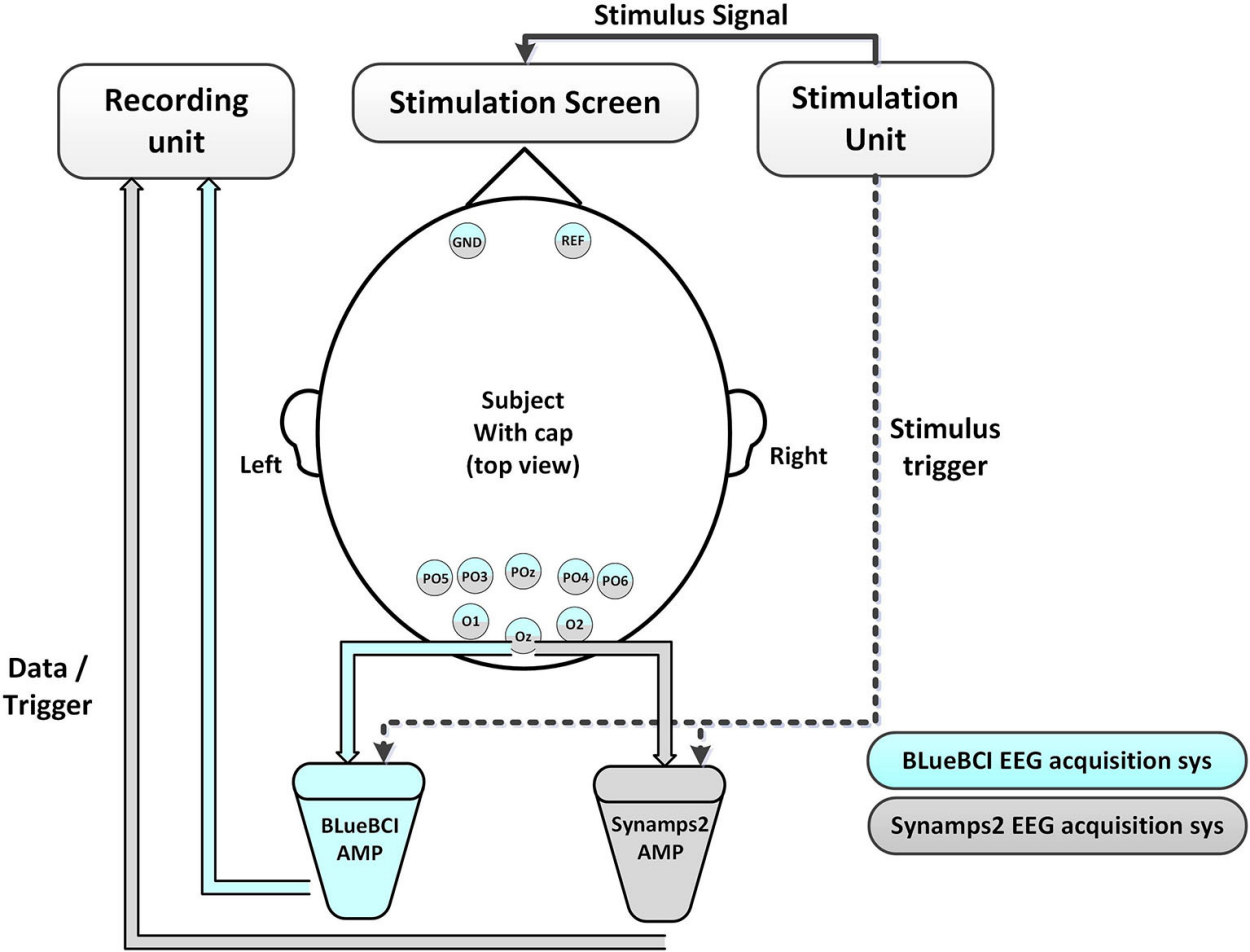
The comparison scheme between BLueBCI and Synamps2 amplifier.

During SSVEP experimentation, recordings of data were independently conducted using both the proposed device and Synamps2 in separate sessions. Initially, subjects wore a wet electrode EEG cap and the impedance was ensured <20 k. The experiment sequence was as follows: initial preparation -> System A -> System B -> Rest -> System B -> System A -> End. In the crossover experiment, we randomly selected the first system. To ensure that the participants remained in a good state throughout the experiment, the total duration could not exceed 90 min. The same stimulation screen, stimulus unit, EEG cap, and trigger sender shown in Figure 5 were used during data acquisition, while different systems were switched via a hardware connector.

In the forty-targets and single-target SSVEP spelling test, EEG data were recorded at a 1,000 Hz sampling rate, using the reference electrode, the ground electrode at the position shown in Figure 5. The Synamps2 amplifier sends raw data/trigger to the recording unit (another host computer) using a wire connection. The BLueBCI amplifier wirelessly sends it to the recording unit via TCP/IP socket. The device in the proposed system is named as BLueBCI.

##### 3.2.2.1. Forty-targets SSVEP online analysis

**Figure 6:**
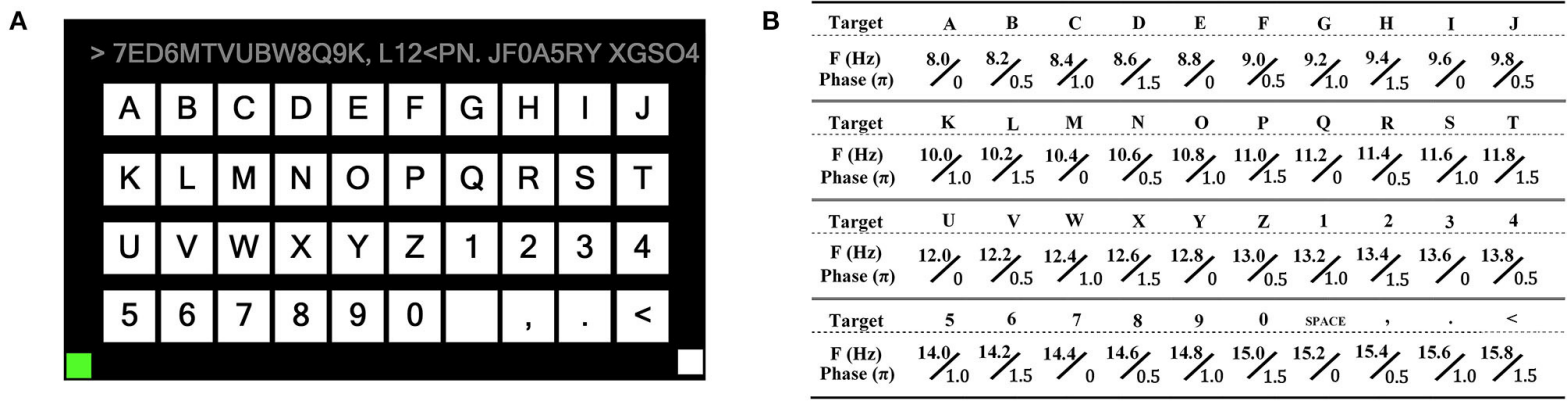
Forty targets of BCI spelling board. **(A)** A board layout with 26 letters, 10 numbers, and four non-alphanumeric keys (space, comma, dot, and backspace) arranged in four rows and 10 columns. The upper is used to display the input characters. **(B)** Encoding the frequency and initial phase of each target using joint frequency and phase modulation (Chen et al., 2014).

**Figure 7:**
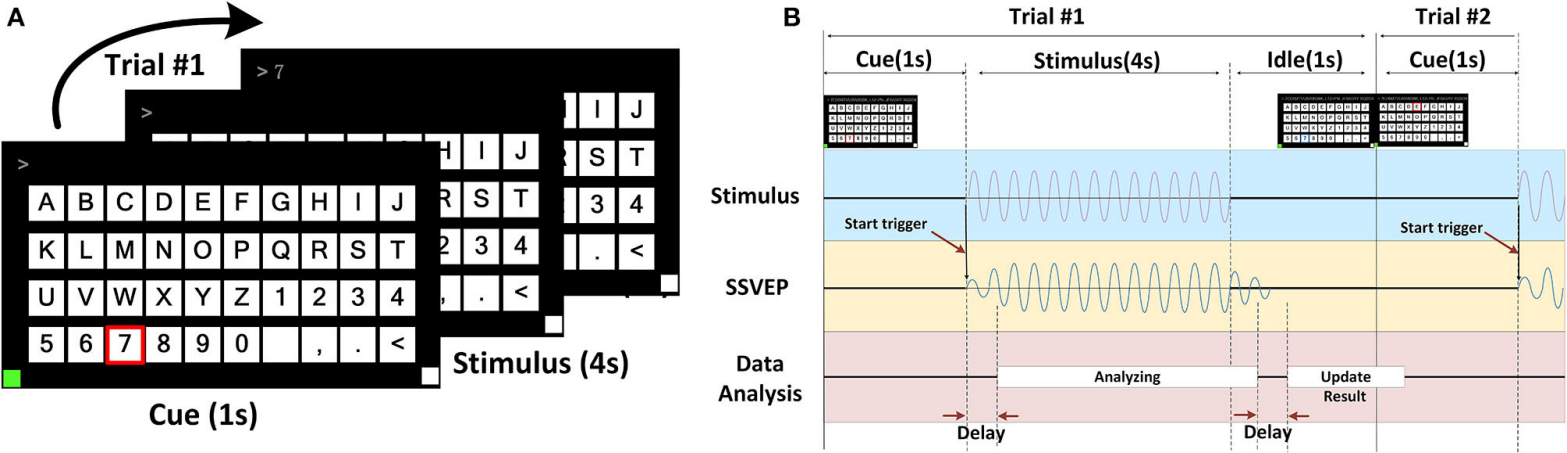
The scheme of SSVEP trail. **(A)** Each block consisted of 40 trails. Each trial was divided into three periods: cue, stimulus, and idle period. The cue was given 1 s (a red box around the target). The subjects followed the cue target, and then, all the targets started flashing at the same time for 4 s. At the end of each trial, the participants had a 1-s idle period. **(B)** Trigger started at the end of the cue and the beginning of the stimulus. During the 4-s flashing, subjects were asked to avoid eye-blinking while flashing. The real-time SSVEP EEG data were analyzed. During the 1-s idle period, the EEG feedback was analyzed using the FBCCA method, and then, the updating result was presented in the input field above, and then, the next trial was followed.

We designed a forty-targets SSVEP spelling board scenario with a display frequency ranging from 8–15.8 Hz, as shown in Figure 6. The scheme of SSVEP trail is shown in Figure 7.

The frequency value of each character in the matrix can be represented:


(6) $$\begin{array}{l} \begin{array}{l} \begin{array}{c} f(k_{x},k_{y})=f_{0}+\text{Δ}f\times [(k_{y}-1)+(k_{x}-1)\times 10],\\ k_{x}\in [1\;\;4],\;k_{y}\in [1\;\;10] \end{array} \end{array} \end{array}$$

where *k*_*x*_ and *k*_*y*_ represents the row index and column index, respectively. In this study, *f*_0_ was 8 Hz and Δ*f* was 0.2 Hz.

We collected all forty-targets SSVEP data from seven healthy subjects (four males and three females, aged 27 ± 5 years). All participants were either students or staff members from the university and were situated 100 cm away from a monitor during the experiment. The participants had a normal or corrected-to-normal vision and had signed consent papers.

##### 3.2.2.2. Single-target SSVEP offline analysis

The forty-targets SSVEP experiment features certain limitations. First, peripheral scintillations are aliased, which affects the subjects' vision and reduces the SNR. This may lead to inaccuracies in the SNR measurements. Second, the low scintillation frequency results in insufficient response at high frequencies. These factors constrain the extent to which the system's performance can be fully evaluated.

A supplementary experiment was carried out. We designed a single-target SSVEP spelling board scenario with frequencies of 12 Hz and 30 Hz. The experimental procedure was similar to the forty-targets experiment. The target was set in a circular area positioned at the center of the screen. As there was only one target, we removed the cue period from each trail. Blocks spanned 75 s and comprised 15 trials, with each trial lasting 4 s of stimulation followed by 1 s of rest.

All single-target SSVEP data were acquired from nine healthy subjects (one male and eight females with an age of 26 ± 1 years). All participants were either students or staff members from the university and were situated 100 cm from the monitor during the experiment. Participants had a normal or corrected-to-normal vision and had signed consent papers.

### 3.3. System evaluation in group cognition task

Mental fatigue was associated with increased power in theta (θ) and parietal alpha (α) EEG rhythms. Sleepiness is typically characterized by an increase in theta and alpha activity, with a decrease in the beta band (Balandong et al., 2018).

In cognitive science, frequency domain features are widely used to assess mental fatigue or sleepiness (Eoh et al., 2005). Brain rhythms are generally divided into five sub-bands: δ : 0.5–4 Hz; θ : 4–8 Hz; α : 8–13 Hz; β : 13–25 Hz; and γ : 25–40 Hz. One of the classic formulas is as follows:


(7) $$\begin{array}{l} F_{1}=\frac{E_{\theta }+E_{\alpha }}{E_{\beta }} \end{array}$$

where the total frequency band power:


(8) $$\begin{array}{l} E=\overset{N_{\text{FFT}}}{\underset{n=1}{\sum }}{\left(\frac{F\left(n\right)}{N_{\text{FFT}}}\right)}^{2} \end{array}$$

where *F*(*n*) denotes the results of the signal X(*n*) at frequency *n*.

**Figure 8:**
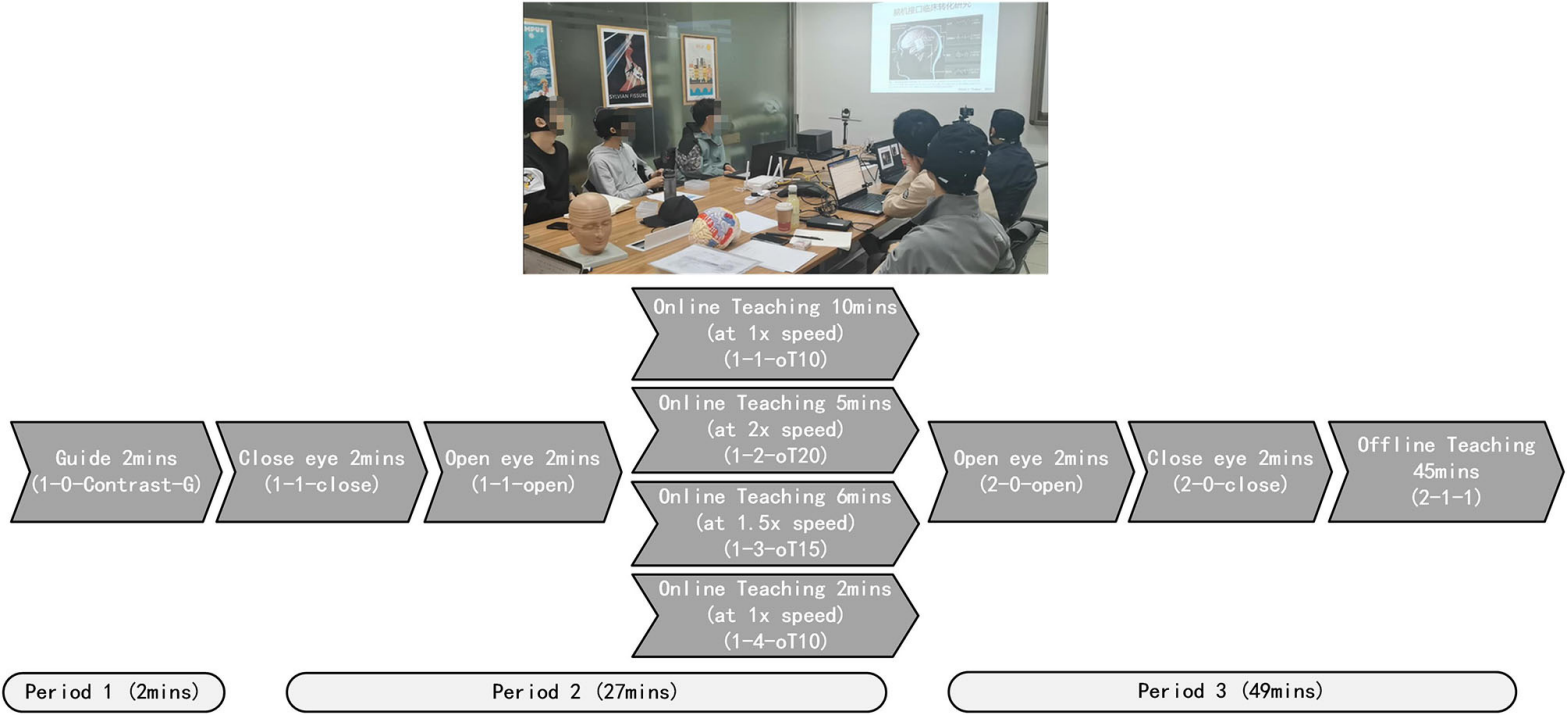
The mBCI scenario for education application.

Quick and reliable signal acquisition is vital for large-scale applications, especially for the classroom cognitive application. To achieve this, we used dry electrodes to collect EEG data from the occipital area in noisy settings. In this group cognition test, the data were acquired from 10 healthy subjects, consisting of nine students and one teacher. The mBCI scenario is shown in Figure 8.

Estimating the mBCI application test was constructed. The teaching process was divided into four parts. This process was as follows: Initially, all subjects prepared EEG cap for 10 min before class, Period 1 (Guide 2 min), Period 2 (Online teaching 27 min), and Period 3 (Offline teaching 49 min), as shown in Figure 8. There was no break from 10:00 to 11:20 AM. Before the online teaching, 2 min of guide (1-0-Contrast-G) was used as the control group. During Period 2, the process included closing the eyes for 2 min (1-1-close), opening the eyes for 2 min (1-1-open), playing the teaching video at 1x speed for 10 min (1-1-oT10), playing the video at 2x speed for 5 min (1-2-oT20), playing the video at 1.5x speed for 6 min (1-3-oT15), and then playing the video at 1x speed for 2 min (1-4-oT10). During Period 3, the process included opening the eyes for 2 min (2-0-open), closing the eyes for 2 min (2-0-close), followed by offline teaching for 45 min (2-1-1).

## 4. Results and discussion

### 4.1. Comparison of synchronization performance

We delivered a light input from an optical sensor and used an oscilloscope to detect two types of delays. One oscilloscope probe is used to test the output of the optical sensor, and the other probe is used to test the TTL signal output from the trigger-receiver unit before entering the microcontroller unit IO port, as shown in Figure 3B.

We measured the time difference between the probes using an oscilloscope. We found a static delay of ~4 ms and a dynamic delay of roughly 0.9 ms over the course of the 30-min recording period, as shown in Figure 3B. The static delay and dynamic delay observed at the edges of the square wave indicated that time drifted from synchronous sampling, as shown in Figure 3B.

This result suggests that the data recorded by the 10 amplifiers were not precisely aligned. The static delay variation is <1 μs, which is almost constant over time. The static delay can be subtracted from the recorded data, while the dynamic delays can potentially lead to errors in the analysis of brain activity below the 1 ms level.

**Figure 9:**
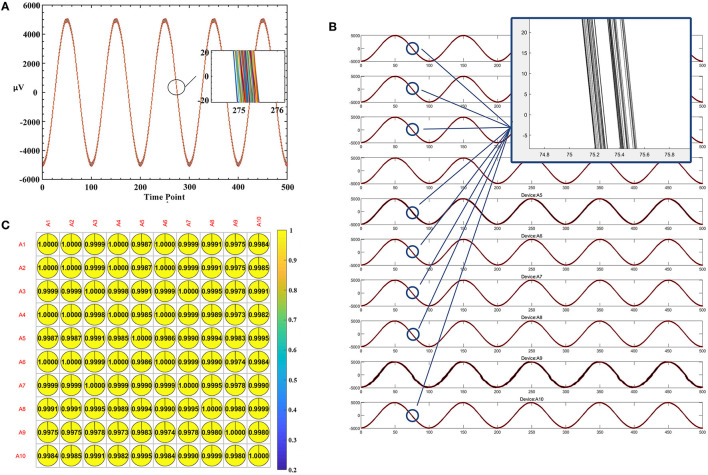
Estimating dynamic delay and correlation. **(A)** The plot of 80 consecutive segments of A1 first channel. **(B)** The plot of eight-channel signals from 10 devices. **(C)** The mutual covariances of each device's first channel.

Furthermore, to assess the synchronization performance, we conducted an experiment to calculate the dynamic delay between the signals. We took the last 50,000 points (1,000 Hz by 50 s) with the trigger and used the Butterworth 50 Hz notch filter. After aligning the trigger, we analyzed the correlation of 40,000 points data. To assess the cumulative phase error of individual device, we have analyzed the signals of one channel. We split the 40,000 points of A1 first channel into 80 consecutive segments (one segment every five cycles, 1 k sample rate, 100 points per cycle). These 80 segments' points are drawn in Figure 9A. The enlarged part of the figure shows that the deviation is in the range of 0.5 ms. To evaluate the phase shift between multiple devices, the signals from 8 channels of 10 devices were analyzed. The signals of 8 channels from 10 devices were plotted simultaneously in Figure 9B. This showed the waves are well synchronized and have a 0.5 ms dynamic delay. The mutual covariances of each device's first channel were shown in Figure 9C. The average correlation reached 99.93%.

After 30 repeated experiments, the minimum synchronization error was 237 μs and the average was 0.9 ms. The causes of the minimum synchronization error would refer to the cumulative “time delay” of the electronic system in the wireless transceiver process, including the baseband protocol resolution time delay, the crystal clock difference of all the subsystems, and micro-control-unit command sequence time difference.

Among all EEG systems, Emotiv is the most commonly used wearable system in research studies (Roy et al., 2019), whereas Brain Products, EGI, BIOSEMI, and g.Tec are the most frequently used desktop systems in hyperscanning studies (Barraza et al., 2019).

In particular, for hyperscanning setup types, a wireless wearable system can overcome limitations on subjects' range of motion and adapt to the experimental paradigm design (Xu et al., 2021). While a wired fixed-linked system is widely used, it can be quite inconvenient and time-consuming when conducting hyperscanning experiments. In addition, it is complicated to acquire simultaneous data with multiple devices. It suggests that wireless wearable setups with a moderate number of channels (8/16/32) can be the most suitable for the mBCI system.

According to our literature review, some related work for the mBCI application are listed in Table 1. Several wired trigger boxes are able to distribute a SYNC signal to different devices simultaneously, performing a hardware-based trigger synchronization method, such as, g.TRIGbox (by g.Tec), USB2 Receiver (by BIOSEMI), and Clock Sync box (by EGI). The systems from Brain Products, EGI and BIOSEMI adopt parallel interfaces of the host computer for hardware-based trigger synchronization since the hardware interrupt level of the parallel interface has a high priority. This can result in a faster response of processing, such as the device from g.Tec, which reaches a synchronization delay of 51.22 ms. Synchronization delay of a wireless system from Emotive adopting audio/video data is 162.69, while the proposed system achieves a far smaller synchronization delay of <1 ms by exploiting light signal and customized data protocol.

**Table 1:** Comparison between the proposed and other commercial EEG acquisition system.

**Devices**	**Brain products^a^**	**EGI^a^**	**BIOSEMI^a^**	**g.Tec g.USBamp^b, c^**	**Emotiv EPOC^c, d^**	**The proposed**
Hyperscanning setup types	Fixed-linked	Fixed-linked	Fixed-linked	Fixed-linked	Wireless wearable	Wireless wearable
Channels number	32/64	128	64	16	14	8/16/32
Device number	2	2	2	4	9	10
Synchronization delay	Not mentioned	Not mentioned	Not mentioned	51.22 ms	162.69 ms	<1 ms
Trigger synchronization method	Wired TTL Software: LSL protocol	Wired TTL Clock Sync box	Wired USB receiver	Wired USB (g.TRIGbox) Software: LSL protocol	Wireless audio/video data Software: LSL protocol	Wireless light signal Software: customized data protocol

^a^By Barraza et al. (2019).

^b^By Bilucaglia et al. (2020).

^c^By Wang et al. (2019).

^d^By Poulsen et al. (2017).

### 4.2. SSVEP performance comparisons

In this section, we present a comparative result of the mBCI devices with temporal and spatial analyses. Each participant had individual REF and GND channels. The channels were connected to the individual amplifiers. In the acquisition software, the EEG data with the triggers can be viewed separately and stored in different files.

The raw SSVEP data underwent several filtering steps. Firstly, we extracted the 4-s stimulated data by the trigger event (the sampling rate of both devices was 1,000 Hz, and 4-s data were 4,000 points per trial). Secondly, any trail data with an amplitude of >100 μV were removed, after which the data were downsampled to 250 Hz. Thirdly, the data were processed by Chebyshev type I bandpass filter, which has a stopband of 3–6 Hz, a passband of 6–65 Hz, and a stopband of 65–75 Hz. This was to eliminate environmental noise. Following data preprocessing, the EEG signal was stored as three-dimensional data with channel × time points × blocks.

#### 4.2.1. Forty-targets SSVEP

**Figure 10:**
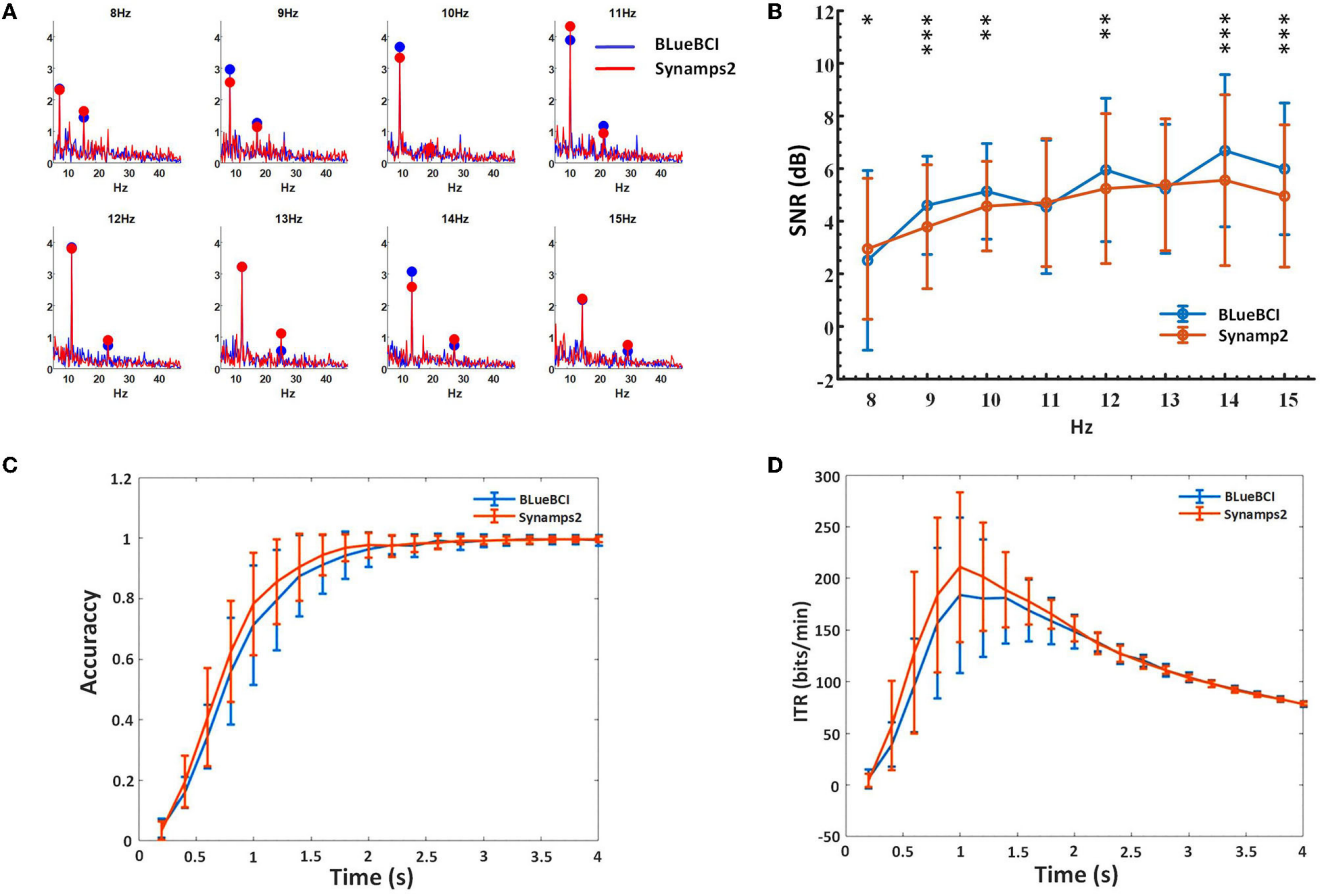
Key performance comparison in the online forty-targets SSVEP experiments. **(A)** Spectrum diagram. **(B)** SNR based on the FBCCA method. The asterisks indicate significant difference by paired t-tests (*p < 0.8, **p < 0.6, ***p < 0.4). **(C)** Classification accuracy. **(D)** ITR. We estimated by data lengths ranging from 0.2 to 4 s, with 0.2-s intervals.

In the forty-targets SSVEP experiment, we focused on the data resulting from the stimulus frequencies of 8, 9, 10, 11, 12, 13, 14, and 15 Hz. The comparison spectrum diagram plot was drawn between the two devices, as shown in Figure 10A. The comparison data between one device of the mBCI system and the Synamps2 device showed obvious SSVEP fundamental frequency and harmonic response.

We compared 11 sets of BLueBCI data and 13 sets of Synamps2 data by analyzing frequencies of 8, 9, 10, 11, 12, 13, 14, and 15 Hz. Paired *t*-tests showed that there was no significant difference in classification accuracy and ITR between the mBCI system device and the Synamps2 device (*p* = 0.820 and 0.656, respectively). However, *t*-tests of SNR showed there was some difference between them, as depicted by the asterisks in Figure 10B.

The results show that the average SNR of BLueBCI (5.08 ± 2.03 dB) is higher than that of Synamps2 (4.66 ± 1.76 dB), as shown in Figure 10B. Based on the 0.2-2 s data, the resulting peak accuracy for BLueBCI is slightly lower than that for Synamps2. With 2 s data length, the accuracy of target recognition was 98%, similar to that of Synamps2 (99%), as shown in Figure 10C. Based on the 0.2-2.2 s data, the ITR of BLueBCI was lower than that of Synamps2. But the ITR performance of the BLueBCI and the Synamps2 tends to be identical after 2.2 s. With 2 s data length, the average ITR reached 150 ± 20 bits/min, and the highest reached 260 bits/min (data length: 1 s), which was comparable to Synamps2 (the average: 150 ± 15 bits/min, the highest: 280 bits/min), as shown in Figure 10D.

#### 4.2.2. Single-target SSVEP

**Figure 11:**
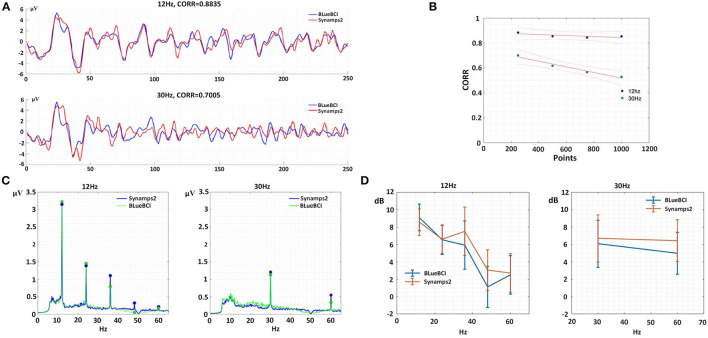
Typical temporal and spatial characteristics. **(A)** Temporal analysis at 250 points per second. **(B)** Unary linear fitting graph of similarity and 1,000 sampling points. **(C)** Spectral analysis, left: fundamental (12 Hz) to fifth harmonic (24, 36, 48, and 60 Hz), right: fundamental (30 Hz) to second harmonic (60 Hz). **(D)** SNR analysis for single-target SSVEP.

In the single-target SSVEP experiment, we obtained averaged data for each device at each frequency from all subjects to eliminate time-space and phase differences caused by multiple subjects. The averaged data were plotted in Figure 11A. Linear fitting was conducted to evaluate the similarity and sampling points as shown in Figure 11B. Firstly, in terms of 12 Hz, the similarity remained nearly constant with an average of 86% as sampling points increased. Secondly, in terms of 30 Hz, the similarity showed a significant negative correlation with the increase in sampling points. This observation suggests that the similarity gradually decreased with increasing frequency, possibly due to insufficient data or multi-subject inconsistent time-space and phase response to a high-frequency stimulus. Our results imply that this approach can be useful for screening individuals for efficient interaction and remain a potential area for further study.

Among the nine subjects, 20 sets of 12 Hz-target and 21 sets of 30 Hz-target were collected by Synamps2; 19 sets of 12 Hz-target and 19 sets of 30 Hz-target were collected by BLueBCI. In Figure 11C, the data by BLueBCI (green line) and Synamps2 (blue line) showed obvious fundamental frequency and harmonic responses, among which the 4th and 5th harmonic responses in Synamps2 12-Hz stimulation were obvious, while the BLueBCI exhibited better performance. However, in the 12-Hz stimulus, the fundamental and second harmonic responses for BLueBCI were higher than that for Synamps2. In the 30-Hz stimulus, the fundamental frequency and second harmonic frequency were lower than that of Synamps2. We compared frequency responses SNR of 12 Hz and 30 Hz, respectively, as shown in Figure 11D. The 12 Hz and 24 Hz SNR of BLueBCI were higher (*p* = 0.230 and 0.847, respectively) than Synamps2. There was no significant difference in the fifth frequency (60 Hz, *p* = 0.856). However, for the 36 Hz and 48 Hz SNR for BLueBCI were significantly lower (*p* = 0.048 and 0.009, respectively) than Synamps2. For the 30-Hz stimulus, the fundamental frequency and the second harmonic response (60 Hz) SNR for Synamps2 were higher (*p* = 0.137 and 0.065, respectively) than those of BLueBCI. This implied that there were some minor defects in acquisition and processing. Simulated IIR filtering was carried out (notch 50 Hz) on the raw Synamps2 data, which revealed a gap in the 36–60 Hz region of the spectrum, confirming the same phenomenon detected in the BLueBCI data. This approach can be a method for assessing hardware performance and be a guide for further optimization and system design.

### 4.3. Group cognition result and discussion

**Figure 12:**
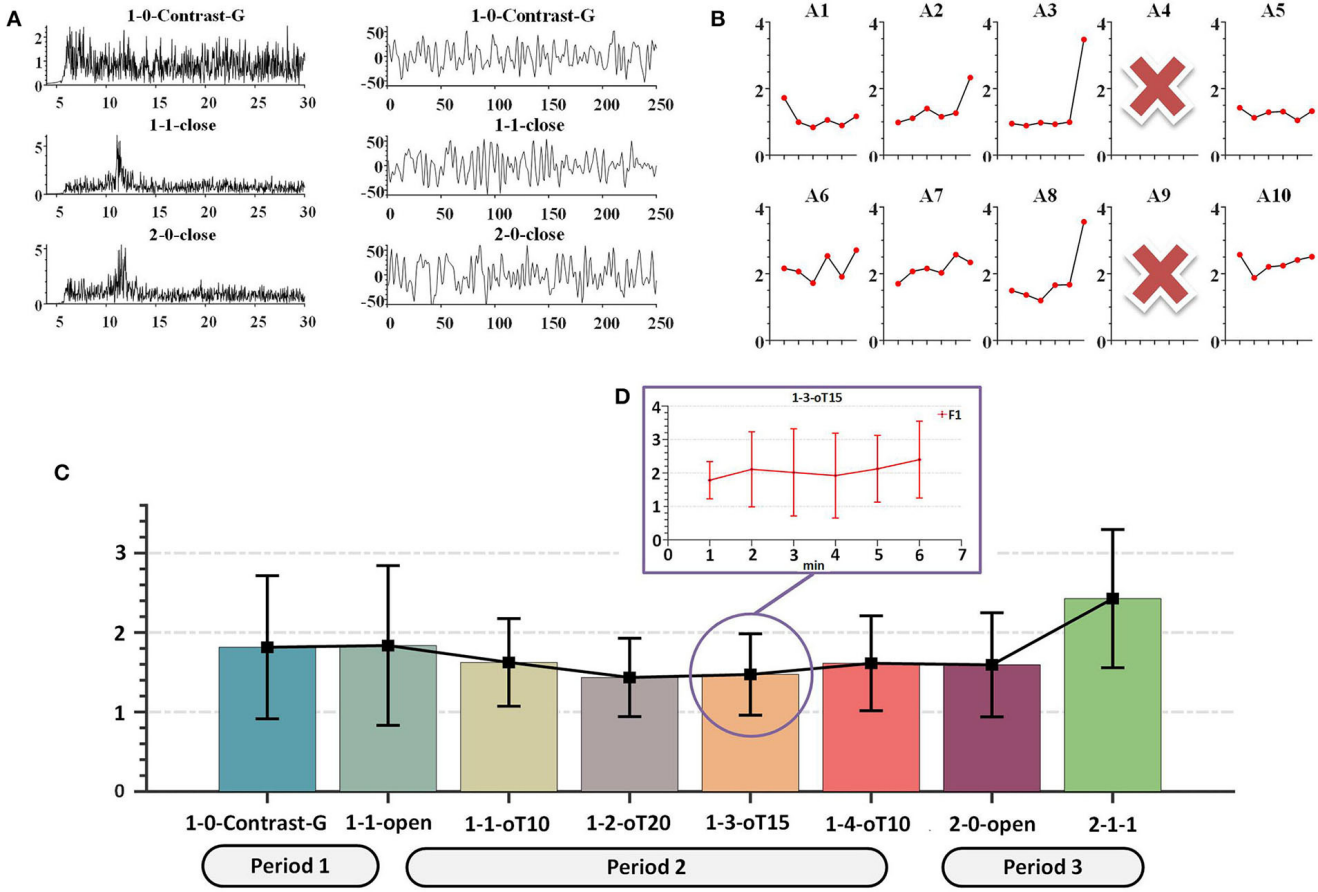
Typical fatigue cognition characteristics. **(A)** Temporal, spatial diagram. **(B)** Each subject F1 During 1-1-oT10 to 2-1-1. **(C)** F1 diagram During 1-0-Contrast-G to 2-1-1, the black line represents the collaborative result. **(D)** During 1-3-oT15, the F1 value with the interval 1 min.

The contrast group data were obtained from the following periods: 1-0-Contrast-G, 1-1-close, and 2-0-close. Both FFT and temporal characteristics were evident in periods 1-1-close and 2-0-close, as depicted in Figure 12A, indicating the validity of the raw data from the quick and simple wearable mBCI system. During the 1-0-Contrast-G period, the fatigue value was used in the performance comparison between online and offline teaching.

The data collected by the A4 and A9 amplifiers were removed due to hardware defects during the evaluation. The data were then downsampled to 250 Hz and filtered by a 50-Hz notch and Chebyshev type I bandpass filter with a passband from 6 to 65 Hz. First, in the hardware performance results, we found that the CMRR was abnormally low and the noise was higher than other devices, as shown in Figure 4 (the device CMRR). Second, due to the abnormal movement of the caps during the experiment, the low and abnormal high-frequency data were filtered.

As shown in Figure 12D, the overall F1 score increased over the 6-min period, indicating a rise in fatigue cognition. Additionally, during the 1-1-oT10 to 2-2-1 stage, there were variations in fatigue levels among subjects, as depicted in Figure 12B. Paired *t*-tests revealed that subjects A1, A5, and A10 showed a significant difference (*p* < 0.03) in fatigue levels compared with the collaborative result, while the fatigue levels of subjects A2, A3, and A8 were consistent (*p* > 0.22) with the collaborative result. Moreover, there was a significant difference between 1-1-oT10 and 2-1-1 periods (*p* = 0.04). The collaborative results showed that the fatigue level decreased during the initial stage of 1-1-open to 1-1-oT20 but subsequently increased from 1-1-oT20 to 2-2-1, as shown in Figure 12C. It revealed an apparent trend of increasing the fatigue level across multiple subjects with personality differences. These outcomes were consistent with the previous fatigue detection research (Eoh et al., 2005).

In future, this wearable mBCI system can be utilized for real-time and rapid multi-subject EEG recording with synchronous collaborative computing. As demonstrated in this multi-subject experiment, it serves as a basic tool for exploring cognitive neuroscience or other multi-subject applications.

## 5. Conclusion

The mBCI system forms the basis for group-cognitive applications. When acquiring brain signals from multiple subjects, it is essential to deploy a wearable, user-friendly, reliable, and sturdy neural recording system with high-performance and synchronization abilities. This cutting-edge wearable mBCI system combines inputs from up to 10 users. First, in terms of SSVEP performance, it results in a higher SNR than NeuroScan Synamps2, with comparable ITR and accuracy. Second, it leverages millisecond-parallel neuro-recording and offers superior portability than other hyperscanning systems. Moreover, the mBCI signal correlation attains 99.8%, with minimal synchronization errors (237 μs). Regarding hardware performance, the average noise amplitude is 0.87 μV, and the average CMRR reaches 109.02 dB. Each wearable compact device weighs just 56 ± 4 g and measures a mere 59.3 × 47.4 × 22.7 mm. In evaluating its suitability for multi-subject teaching applications, preparation required <10 min. Group-cognitive assessment findings not only reveal individual variations but also offer insights into group EEG fatigue cognitive neurology.

Evaluation results indicate that the proposed mBCI system is a highly efficient tool for real-time research and the system will facilitate various applications in the fields of swarm intelligence and cognitive neurology.

## Data availability statement

The raw data supporting the conclusions of this article will be made available by the authors, without undue reservation.

## Ethics statement

The studies involving human participants were reviewed and approved by the Institution Review Board of Tsinghua University. The patients/participants provided their written informed consent to participate in this study.

## Author contributions

YoH, YW, and LZ: study conception and design. YoH: data collection and visualization. YW, WP, and XG: experiment validation. YW, ZZ, and LZ: writing—review and editing. YoH and YuH: analysis and interpretation of results. LZ: supervision and funding acquisition. All authors contributed to the article and approved the submitted version.
